# Distinct Epithelial Cell Profiles in Normal Versus Induced-Congenital Diaphragmatic Hernia Fetal Lungs

**DOI:** 10.3389/fped.2022.836591

**Published:** 2022-05-06

**Authors:** Ana N. Gonçalves, Jorge Correia-Pinto, Cristina Nogueira-Silva

**Affiliations:** ^1^Life and Health Sciences Research Institute (ICVS), School of Medicine, University of Minho, Braga, Portugal; ^2^ICVS/3B’s - PT Government Associate Laboratory, Guimarães, Portugal; ^3^Department of Pediatric Surgery, Hospital de Braga, Braga, Portugal; ^4^Department of Obstetrics and Gynecology, Hospital de Braga, Braga, Portugal

**Keywords:** alveolar type 2 cell (AEC2), CDH, ciliated cell, clara cell, PNECs

## Abstract

**Background:**

Recent studies identified a great diversity of cell types in precise number and position to create the architectural features of the lung that ventilation and respiration at birth depend on. With damaged respiratory function at birth, congenital diaphragmatic hernia (CDH) is one of the more severe causes of fetal lung hypoplasia with unspecified cellular dynamics.

**Objectives:**

To characterize the epithelial cell tissue in hypoplastic lungs, a careful analysis regarding pulmonary morphology and epithelial cell profile was conducted from pseudoglandular-to-saccular phases in normal versus nitrofen-induced CDH rat lungs.

**Design:**

Our analysis comprises three experimental groups, control, nitrofen (NF) and CDH, in which the relative expression levels (western blot) by group and developmental stage were analyzed in whole lung. Spatiotemporal distribution (immunohistochemistry) was revealed by pulmonary structure during normal and hypoplastic fetal lung development. Surfactant protein-C (SP-C), calcitonin gene-related peptide (CGRP), clara cell secretory protein (CCSP), and forkhead box J1 (FOXJ1) were the used molecular markers for alveolar epithelial cell type 2 (AEC2), pulmonary neuroendocrine, clara, and ciliated cell profiles, respectively.

**Results:**

Generally, we identified an aberrant expression of SP-C, CGRP, CCSP, and FOXJ1 in nitrofen-exposed lungs. For instance, the overexpression of FOXJ1 and CGRP in primordia of bronchiole defined the pseudoglandular stage in CDH lungs, whereas the increased expression of CGRP in bronchi; FOXJ1 and CGRP in terminal bronchiole; and SP-C in BADJ classified the canalicular and saccular stages in hypoplastic lungs. We also described higher expression levels in NF than CDH or control groups for both FOXJ1 in bronchi, terminal bronchiole and BADJ at canalicular stage, and SP-C in bronchi and terminal bronchiole at canalicular and saccular stages. Finally, we report an unexpected expression of FOXJ1 in BADJ at canalicular and saccular stages, whereas the multi cilia observed in bronchi were notably absent at embryonic day 21.5 in induced-CDH lungs.

**Conclusion:**

The recognized alterations in the epithelial cell profile contribute to a better understanding of neonatal respiratory insufficiency in induced-CDH lungs and indicate a problem in the epithelial cell differentiation in hypoplastic lungs.

## Introduction

Respiratory function is dependent on lung architecture, created and maintained by interactions of myriad cells during gestational life. Importantly, the traditional view of fetal lung development subdivides the lung morphogenesis into five distinct periods based on structure: embryonic, pseudoglandular, canalicular, saccular, and alveolar periods, which are shared among mammalian species [reviewed in Refs. (1, 2)]. At the molecular level, it is the expression of *Nkx2-1* in the endoderm of the ventral wall of the anterior foregut that first identified the lung at the embryonic stage (3). Afterward, mesodermal-endodermal interactions support branching morphogenesis and the specification of multipotent progenitor cells into proximal (SOX2) versus distal (SOX9) profiles (4–11), reviewed in Ref. (12). Interestingly, the differentiation of proximodistal patterning at the time of conducting and respiratory airways formation contribute for normal respiratory function at birth. More relevant, the current knowledge of epithelial cell differentiation admits distinct models for bronchiolar (SOX2^+^) versus alveolar (SOX9^+^) lineages, in which the bronchiolar differentiation gives rise to goblet, clara, ciliated, and neuroendocrine cells under mechanisms dependent on Notch signaling (13–18), whereas SOX9 or a region just proximal to SOX9^+^ cells at early or a bipotent progenitor at later developmental stage form alveolar epithelial cell type 1 and 2 (AEC1 and AEC2) [Frank et al. (19), Desai et al. (20), and Treutlein et al. (21)]. AEC1 cells constitute about 95% of the surface area and are located immediately adjacent to the capillaries, which allows efficient O_2_ and CO_2_ diffusion, while AEC2 cells secrete surfactants to prevent alveolar collapse (20, 22).

Reaching its maximum severity in the congenital diaphragmatic hernia (CDH), fetal lung hypoplasia remains as one of the most common causes of morbidity and mortality for neonates. CDH is defined as a diaphragmatic defect that allows the herniation of abdominal organs into the thorax and impairs the normal fetal lung development (23, 24). Hypoplastic lungs have reduced surface area for gas exchange, with a decrease in distal branching and alveoli. The alveoli that do exist have thicker walls, impairing the close association of the airspaces to the capillaries (25–27). A recent publication has shown the proximodistal patterning impaired in induced-CDH lungs from pseudoglandular-to-saccular stages (28), whereas the epithelial cell dynamics, resulting from those differentiation continues uncertain. In this context, taking advantage of the nitrofen-induced CDH rat model that mimics the *in vivo* human CDH in terms of the disrupted signal pathways in branching morphogenesis and alveolar differentiation (29), we performed a careful analysis regarding the pulmonary morphology and the epithelial cell profiles during normal versus hypoplastic pulmonary development.

## Materials and Methods

This study was carried out in strict accordance with FELASA guidelines (30) and European regulations (European Union Directive 86/609/EEC). All animal experiments were approved by the Life and Health Sciences Research Institute (ICVS), University of Minho, and by the Direção Geral de Alimentação e Veterinária (approval No. DGAV 021328).

### Animal Model and Experimental Design

Sprague-Dawley female rats (225 g; Charles-River, Spain) were maintained in appropriate cages under temperature-controlled room (22–23°C) on 12 h light: 12 h dark cycle, with commercial solid food and water *ad libitum*. The rats were mated and checked daily for vaginal plug. The day of plugging was defined as embryonic day (E) 0.5 for time dating purposes. According to the nitrofen-induced CDH rat model (31, 32), at E9.5, randomly selected pregnant rats were exposed to 100 mg nitrofen (2,4-dichlorophenyl-*p*-nitrophenylether). At different time points (E17.5, E19.5, and E21.5), fetuses were harvested by cesarean section. After fetal decapitation, a thoracic laparotomy was performed under a binocular surgical microscope (Leica Biosystems, Wild M651.MSD, Washington, United States) to inspect the diaphragm and harvest the organs. Fetuses were divided into three groups, namely the control (Ctrl), fetuses exposed to olive oil alone; nitrofen (NF), fetuses exposed to nitrofen without diaphragmatic defect; and CDH group, fetuses exposed to nitrofen with diaphragmatic defect. Lungs were either fixed in 4% paraformaldehyde for immunohistochemistry or snap-frozen in liquid nitrogen for protein extraction. GPower 3.1.9.4 (Franz Faul, Universitat Kiel, Germany) was used for sample size calculation. In total, 18 dams and 124 embryonic rats were used in this study.

### Immunohistochemistry

As previously described (33), immunostaining was performed in formalin-fixed and paraffin-embedded sections at different gestational ages (E17.5–E21.5) for the three groups, control, NF, and CDH. Primary antibodies for alveolar epithelial cell type 2 (AEC2, Anti-Prosurfactant Protein C, SP-C, 1:1,000, Cat No. AB3786, Merck Millipore, Germany); Clara (Anti-Clara Cell Secretory Protein, CCSP, 1:1,000, Cat No. 07-623, Merck Millipore, Germany); ciliated (FOXJ1, 1:200, Cat No. PA5-36210, ThermoFisher Scientific, Massachusetts, United States); pulmonary neuroendocrine cells/neuroepithelial bodies (PNECs/NEBs; 1:100, CGRP, Cat No. ab91007, Abcam, Cambridge, United Kingdom) were used. Negative control reactions included omission of primary antibody, in which immunoreactivity was not observed. Tissue sections were incubated with a streptavidin-biotin immunoenzymatic antigen detection system (Cat No. TL-125-QHD, Thermo Scientific, Massachusetts, United States) according to the manufacturer’s instructions and visualized with a diaminobenzidine tetrahydrochloride solution (DAB, Cat No. TA-125-QHDX, Thermo Scientific, Massachusetts, United States) (33). The time expended in DAB solution was dependent on the developmental stage, but equally between normal, NF and CDH slides, allowing the quantification of immunohistochemical signals. The percentage of stained cells per microscopic field was scored as follows: 0, 0–1% cells/pulmonary structure; 1, 1–25% cells/pulmonary structure; 2, 25–50% cells/pulmonary structure; 3, 50–75% cells/pulmonary structure; 4, 75–100% cells/pulmonary structure in accordance with (28). At least three independent experiments were performed for each antibody tested, comprising different and unrepeated animal samples by group (gestational age). Six different animals were examined for each group per studied antibody. All sections were scanned with Olympus BX61 Upright Microscope (Olympus Corporation, Tokyo, Japan) and independently evaluated by two investigators.

### Western Blot Analysis

Normal and nitrofen-exposed lungs from different gestational ages (E17.5–E21.5) were processed for western blot analysis. Proteins were obtained according to Ref. (34), and the protocol performed as previously described (28). Blots were blocked in 5% bovine serum albumin and probed with primary antibodies for AEC2 (Anti-Prosurfactant protein-C, SP-C, 1:500, Cat No. AB3786); clara (clara cell secretory protein, CCSP, 1:500, Cat No. 07-623); ciliated (FOXJ1, 1: 100, Cat No. PA5-36210); PNECs/NEBs (CGRP, 1:250, Cat No. ab91007) according to the manufacturer’s instructions. For loading control, blots were probed with β-tubulin (1:200,000, Cat No. ab6046 Abcam, Cambridge, United Kingdom). Membranes were then incubated with anti-rabbit secondary horseradish peroxidase-conjugate (1:5,000, Cat No. 7074, Cell Signaling, Technology, Massachusetts, United States), developed with Clarity West ECL subtract, and the chemiluminescent signal was captured using the Chemidoc XRS. The quantitative analysis was performed with Quantity One 4.6.5 1-D Analysis Software. Three independent experiments were performed (*n* = 3). In total, nine animals were used in each group (gestational age/condition) per antibody.

### Statistical Analysis

All quantitative data are presented as the mean ± standard deviation (SD). The statistical analysis was performed by two-way ANOVA for lung condition (normal, NF and CDH) and embryonic day (E17.5, E19.5, and E21.5) in protein expression level. The parametric test assumptions were previously verified, and an additional Fisher’s Least Significant Difference (LSD) test was used for post-test analysis. *T*-test for independent samples was performed to compare the molecular spatiotemporal distribution by pulmonary structure and developmental stage. Statistical analysis was performed using the statistical software IBM SPSS Statistics 24.0. Statistical significance was set at ^α^*p* < 0.05.

## Results

**FIGURE 1:**
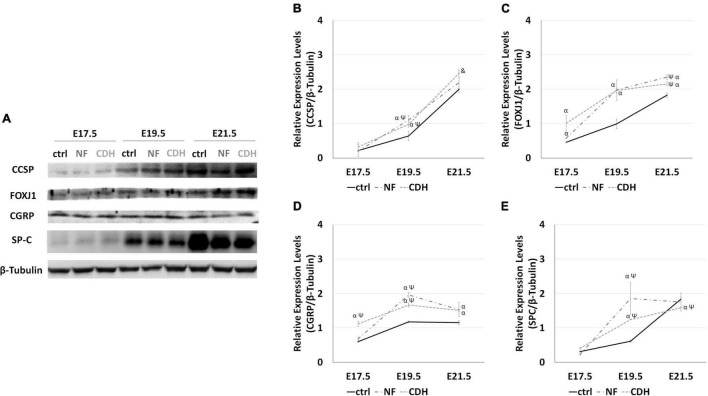
Altered relative expression levels for clara cell secretory protein (CCSP), forkhead box J1 (FOXJ1), calcitonin gene-related peptide (CGRP), and surfactant protein-C (SP-C) in hypoplastic rat lungs. Western blot analysis for CCSP, FOXJ1, CGRP, and SP-C protein levels in normal (ctrl) and CDH lungs at embryonic day (E)17.5-to-E21.5. **(A)** Representative immunoblots are shown. Each lane represents a pooled tissue sample, and relative expression was determined against β-tubulin. Semi-quantitative analysis of three independent experiments is plotted (*n* = 9 per timepoint and experimental groups, respectively). Protein expression levels of **(B)** CCSP, **(C)** FOXJ1, **(D)** CGRP, and **(E)** SP-C are shown at the distinct developmental stages of normal, nitrofen (NF), and congenital diaphragmatic hernia (CDH) fetal lungs. Results are presented as mean ± SD. Symbols indicate the main effects and non-redundant interactions of the two-way ANOVA. *p* < 0.05: ^α^vs ctrl; ^Ψ^vs E17.5-ctrl; ^&^vs E17.5-CDH and E19.5-CDH.

To reveal the epithelial cell profile in hypoplastic lungs, we selected the teratogenic model to induce fetal lung hypoplasia. Nitrofen-induced CDH rat model cause lung hypoplasia with and without CDH; though its severity was greater in those with CDH. As such, a careful analysis regarding the distinct epithelial cells in terms of relative expression levels (Figures 1A–E) and spatiotemporal distribution was performed from E17.5 to E21.5, in which CCSP, FOXJ1, CGRP, and SP-C were used to distinguish Clara, Ciliated, PNECs/NEBs, and AEC2 cellular profiles, respectively.

### Experimental-Congenital Diaphragmatic Hernia Change the Relative Expression Levels of Bronchiolar and Alveolar Markers

In the normal whole lung, quantification of the relative expression levels reveals a consistent increase in CCSP, FOXJ1, and SP-C expression as fetal lung development progresses (Figures 1A–E). Comparing with normal lungs, nitrofen-exposed lungs were characterized by the overexpression of FOXJ1 (Figure 1C) and CGRP (Figure 1D) from pseudoglandular-to-saccular stages. Unchanged expression levels for CCSP (Figure 1B) and SP-C (Figure 1E) were visualized at E17.5, whereas at canalicular stage we identified an increased expression of CCSP and SP-C in hypoplastic (NF and CDH) versus normal lungs. At E21.5, CCSP remains overexpressed, while a slight depletion on SP-C was observed in induced-CDH lungs (Figures 1B,E).

These molecular changes were further explored in terms of spatiotemporal distribution in NF and CDH versus normal lungs. Concomitant with the developmental stage, this analysis also reveals the expression profile by pulmonary structure.

### Similar Spatiotemporal Distribution for Clara Cell Secretory Protein in Nitrofen and Congenital Diaphragmatic Hernia Lungs at E21.5

**FIGURE 2:**
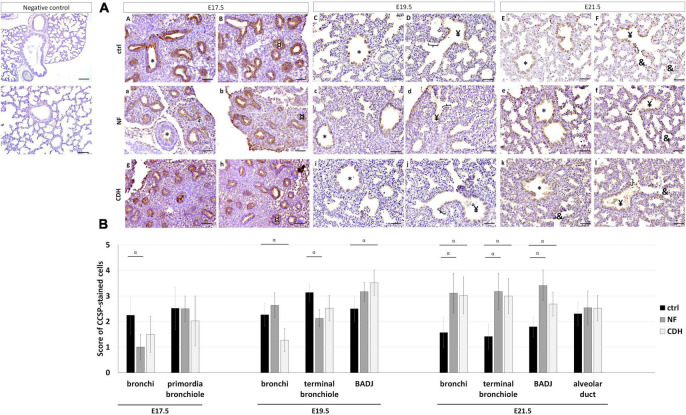
Clara cell secretory protein (CCSP) expression pattern during normal and hypoplastic fetal lung development. Representative immunohistochemical evidence for CCSP expression at **(AA,B,a,b,g,h)** pseudoglandular, **(AC,D,c,d,i,j)** canalicular, and **(AE,F,e,f,k,l)** saccular stages in normal (ctrl), nitrofen (NF), and congenital diaphragmatic hernia (CDH) lungs, respectively. *Bronchiole; ^¤^primordia of bronchiole; *^yen^*terminal bronchiole; [bronchioalveolar duct junction; ^&^alveolar duct. Scale bar 50 μM. **(B)** Semi-quantitative analysis of CCSP expression from embryonic day **(E)** 17.5-to-E21.5 in normal, NF, and CDH lungs. Data are presented as mean ± SD. Symbols indicate the main effects and non-redundant interactions of *T*-test for independent samples. ^α^*p* < 0.05.

Clara cell secretory protein (CCSP) was expressed in all pulmonary structures from pseudoglandular-to-saccular stages in normal and hypoplastic fetal lungs (Figures 2AA–F,a–l). Specifically, CCSP was observed in bronchi and primordia of bronchiole at E17.5 (Figures 2AA,B,a,b,g,h); and in bronchi, terminal bronchiole, and bronchioalveolar duct junction (BADJ) at canalicular (Figures 2AC,D,c,d,i,j) and saccular stages (Figures 2AE,F,e,f,k,l). CCSP + cells were also detected in alveolar duct at E21.5 in normal (Figures 2AE,F), NF (Figures 2Ae,f) and CDH lungs (Figures 2Ak,l).

Quantification of IHC signals by pulmonary structure and developmental stage demonstrated CCSP to be downregulated in bronchi at E17.5 and terminal bronchiole at E19.5 in NF versus control group. In contrast, CCSP was decreased in bronchi and overexpressed in BADJ at E19.5 in CDH versus normal lungs (Figure 2B). A similar expression profile for NF and CDH versus control lungs was observed at E21.5 with the overexpression of CCSP in bronchi, terminal bronchiole, and BADJ (Figure 2B).

### Forkhead Box J1 Expressed in Bronchioalveolar Duct Junction at Canalicular and Saccular Stages After Congenital Diaphragmatic Hernia Induction

**FIGURE 3:**
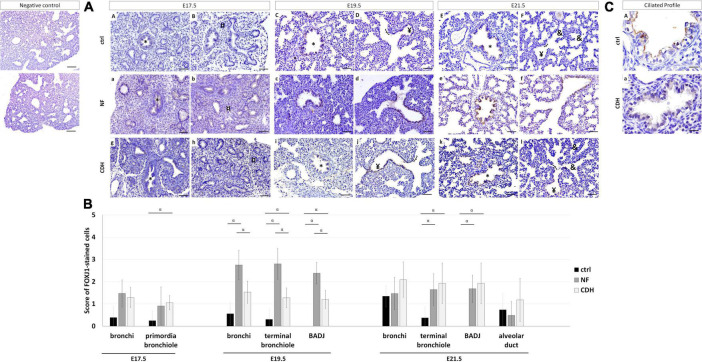
Spatiotemporal distribution of forkhead box J1 (FOXJ1) in normal and induced-congenital diaphragmatic hernia (CDH) rat model at pseudoglandular, canalicular, and saccular stages. Representative immunohistochemical evidence for FOXJ1 expression at **(AA,B,a,b,g,h)** embryonic day (E)17.5, **(AC,D,c,d,i,j)** E19.5, and **(AE,F,e,f,k,l)** E21.5 in normal (ctrl), nitrofen (NF), and CDH lungs, respectively. *Bronchiole; ^¤^primordia of bronchiole; ^¥^terminal bronchiole; [bronchioalveolar duct junction; ^&^alveolar duct. Scale bar 50 μM. **(B)** Semi-quantitative analysis of FOXJ1 expression from pseudoglandular-to-saccular stages in ctrl, NF, and CDH lungs. Results are presented as mean ± SD. Symbols indicate the main effects and non-redundant interactions of *T*-test for independent samples. ^α^*p* < 0.05. **(C)** Representative immunohistochemical evidence for the absence of multi-cilia on the plasma membrane of ciliated cells in bronchi at E21.5. Original magnification ×600.

Forkhead box J1 (FOXJ1) was used to distinguish the ciliated profile in normal and hypoplastic fetal lungs. FOXJ1 was expressed in bronchi (Figures 3AA–F) at all gestational ages; in primordia of bronchiole at E17.5 (Figures 3AA,B); and terminal bronchiole at E19.5 (Figures 3AC,D) and E21.5 (Figures 3AE,F). In hypoplastic (NF and CDH) lungs, FOXJ1 was observed in BADJ at E19.5 (Figures 3Ac,d,i,j) and E21.5 (Figures 3Ae,f,k,l) that contrast with their absence in normal lungs (Figures 3AC–F). In addition, the multi cilia cells observed in bronchi at E21.5 in healthy lungs was demonstrated to be (near) absence in CDH lungs (Figures 3CA,a).

Quantification of IHC signals established FOXJ1 overexpressed in primordia of bronchiole at E17.5 in CDH and bronchi at E19.5 in NF versus normal lungs. Both NF and CDH lungs had increased FOXJ1 expression in terminal bronchiole at E19.5 and E21.5, when compared with normal lungs (Figure 3B). At E19.5, the molecular levels of FOXJ1 in bronchi, terminal bronchiole, and BADJ were higher in NF than CDH or control groups (Figure 3B).

### The Size of Neuroepithelial Bodies Increased in Induced-Congenital Diaphragmatic Hernia Lungs at Canalicular and Saccular Stages

**FIGURE 4:**
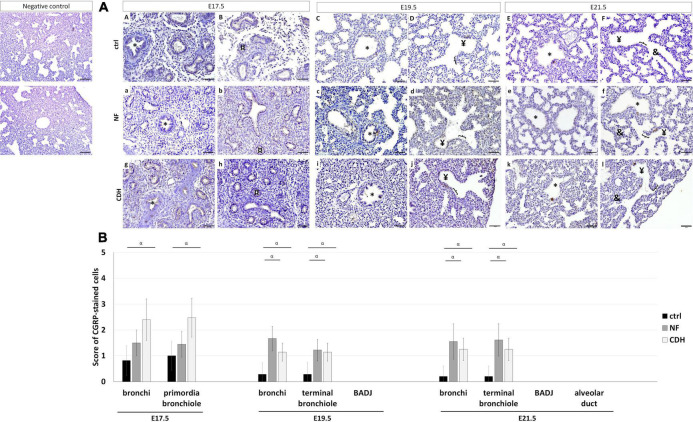
Protein expression pattern of calcitonin gene-related peptide (CGRP) in nitrofen-exposed rat lungs at embryonic day (E) 17.5-to-E21.5. Representative immunohistochemical evidence for CGRP expression at **(AA,B,a,b,g,h)** pseudoglandular, **(AC,D,c,d,i,j)** canalicular, and **(AE,F,e,f,k,l)** saccular stages in normal (ctrl), nitrofen (NF), and congenital diaphragmatic hernia (CDH) rat lungs, respectively. *Bronchiole; ^¤^primordia of bronchiole; ^¥^terminal bronchiole; [bronchioalveolar duct junction; ^&^alveolar duct. Scale bar 50 μM. **(B)** Semi-quantitative analysis of CGRP expression from E17.5-to-E21.5 in normal, NF, and CDH lungs. Results are presented as mean μ SD. Symbol indicates main effect and non-redundant interaction of *T*-test for independent samples. ^α^*p* < 0.05.

Punctual (PNECs) or aggregated (NEBs) expression of CGRP characterize the neuroendocrine profile in the developing lung. Immunohistochemistry analysis showed CGRP expressed in bronchi at E17.5–E21.5; primordia of bronchiole at E17.5 (Figures 4AA,B,a,b,g,h); and terminal bronchiole at E19.5 (Figures 4AC,D,c,d,i,j) and E21.5 (Figures 4AE,F,e,f,k,l) in normal and hypoplastic fetal lungs.

Comparing NF and CDH with normal lungs, CGRP was overexpressed in bronchi and terminal bronchiole at E19.5 and E21.5 in hypoplastic lungs, whereas the significative overexpression in bronchi and primordia of bronchiole at E17.5 was only observed in CDH lungs (Figure 4B). CGRP overexpressed at E19.5 and E21.5 was evidenced by larger NEBs (Figures 4AC–F,c–f,i–l).

### Experimental Congenital Diaphragmatic Hernia Induce the Expression of Surfactant Protein-C in Bronchi and Bronchioalveolar Duct Junction

**FIGURE 5:**
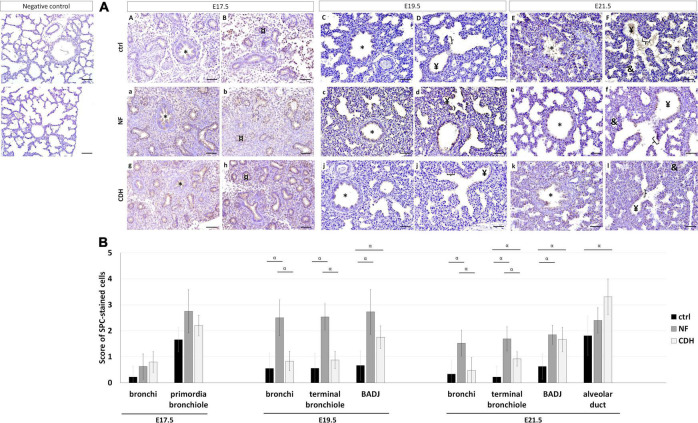
Expression profile of surfactant protein-C (SP-C) in the normal and induced congenital diaphragmatic hernia (CDH) rat model. Representative immunohistochemical evidence for SP-C expression at **(AA,B,a,b,g,h)** embryonic day, (E)17.5, **(AC,D,c,d,i,j)** E19.5, and **(AE,F,e,f,k,l)** E21.5 of normal (ctrl), nitrofen (NF), and CDH phenotypes, respectively. *Bronchiole; ^¤^primordia of bronchiole; ^¥^terminal bronchiole; [bronchioalveolar duct junction; ^&^alveolar duct. Scale bar 50 μM. **(B)** Semi-quantitative analysis of SP-C expression from pseudoglandular-to-saccular stages in control, NF, and CDH lungs. Results are presented as mean μ SD. Symbols indicate the main effects and non-redundant interactions of *T*-test for independent samples. ^α^*p* < 0.05.

The spatiotemporal profile of AEC2 cells was detected by SP-C. In normal and hypoplastic lungs, SP-C was expressed in bronchi at E17.5–E21.5 (Figures 5AA–F,a–f,g–l); in primordia of bronchiole at E17.5 (Figures 5AA,B,a,b,g,h); in terminal bronchiole and BADJ at E19.5 (Figures 5AC,D,c,d,i,j) and E21.5 (Figures 5AE,F,e,f,k,l); and in alveolar duct at E21.5 (Figures 5AE,F,e,f,k,l).

Semi-quantitative analysis of SP-C expression in nitrofen-exposed versus normal lungs showed SP-C overexpressed in BADJ at E19.5 and E21.5, and in terminal bronchiole at E21.5. In induced-CDH lungs, SP-C was also overexpressed in alveolar duct at E21.5 (Figure 5B). By group, the expression of SP-C was increased in bronchi at E19.5 and E21.5 in NF versus normal lungs. Finally, significant differences in bronchi and terminal bronchiole were visualized at E19.5 and E21.5 with SP-C more expressed in NF than CDH or normal lungs (Figure 5B).

## Discussion

Single-cell transcriptomic and tracing-lineage studies allowed the observation of precise number and position of distinct pulmonary cell types, their lineages, and differentiation (35–38). CDH fetuses with decreased distal branching and alveoli manifest reduced respiratory function at birth (39, 40). Recently, the proximodistal patterning was described as impaired in nitrofen-induced CDH lungs from pseudoglandular-to-saccular stages (28). As such, we intend to go further and determine the relative expression levels and the temporospatial distribution for CCSP, CGRP, FOXJ1, and SP-C proteins in hypoplastic (NF and CDH) versus normal fetal lungs from pseudoglandular-to-saccular stages. The selected molecular markers: CCSP, CGRP, FOXJ1, and SP-C identified clara, PNECs/NEBs, ciliated and AEC2 cells, respectively, when expressed in differentiated epithelial tissues. Conversely, when detected in undifferentiated epithelial tissues, they distinguish the cellular capacity to give rise to the above-mentioned epithelial cell types. At the pseudoglandular stage, our findings demonstrated FOXJ1 and CGRP overexpressed in primordia of bronchiole after CDH-induction. As the epithelial cell differentiation goes through, we identified a general overexpression of CGRP in bronchi; FOXJ1, and CGRP in terminal bronchiole; and CCSP and SP-C in BADJ at both canalicular and saccular stages in induced CDH-lungs. Interestingly, in bronchi and terminal bronchiole, CCSP is decreased at canalicular and overexpressed at saccular stages.

Discussing these results, it must be knowledge the distinct contribution of the epithelial progenitors and specialized epithelial cells that populate conducting and respiratory airways. In fact, several studies tried to describe the function of the distinct epithelial cell types during the development of the lung and at birth, when baby takes the first breath. PNECs/NEBs are described as airway sensors required for appropriate innate immune inflammatory response and fetal lung growth. Subsequently, we and others demonstrate PNECs/NEBs overexpressed in *in vivo* nitrofen-exposed lungs, whereas the exogenous administration of neuroendocrine products, like bombesin or ghrelin, stimulate fetal lung growth (38, 41–46). Clara is a secretory cell essential for airway epithelium reparation (47, 48), that it is now described with distinct profiles in NF and CDH lungs. For instance, compared with normal lungs, the expression of CCSP is decreased in bronchi at E19.5, and increased in bronchi and terminal bronchiole at E21.5 and in BADJ at canalicular and saccular stages in CDH. Concerning the NF group, CCSP was decreased in bronchi at E17.5 and terminal bronchiole at E19.5, when compared with control. Ciliated cells are reported as terminally differentiated epithelial cells (49) working in mucociliary clearance at birth and thereafter (50). Now, in hypoplastic lungs (NF and CDH groups), we detected FOXJ1 expressed in BADJ at E19.5 and E21.5 in opposition to the observed in normal lungs. Interestingly, BADJ is formed and easily detected at canalicular stage (51, 52) that demarcates airway-fated epithelial cells from alveolar-fated epithelial cells and works as stem cell niche in adult lung regeneration (53, 54). Indeed, BADJ represents the entrance of the small gas exchanging airways, with critical roles in the formation of both conducting and respiratory airways after injury (53, 54). Our investigation also described FOXJ1 in bronchi, terminal bronchiole, and BADJ as higher in NF than CDH or normal lungs at E19.5. More relevant, we demonstrated the multi cilia on the plasma membrane that characterize a normal bronchus at E21.5 as decrease in induced-CDH lungs. FOXJ1 is a master regulator of basal body docking, cilia formation, and motility (55, 56), whereas the multi cilia on the plasma membrane unequivocally identified their differentiated profile. Together, our observations describe a diffuse transition from conducting to respiratory airways in induced-CDH lungs and suggest an undifferentiated epithelium in hypoplastic lungs.

Epithelial cell type 2 (AEC2) cells produce pulmonary surfactant proteins that reduce the alveolar surfactant tension and facilitate the first breath at birth. In nitrofen-exposed lungs, the impairment on surfactant production and secretion is evidenced by the low levels of phosphatidylcholine, the lipid component of surfactant, and the factors involved in stimulating the maturation of surfactant lipids, such as PTHrP, adipose differentiation-related protein (ADRP), Thy1 and RA, whereas the inhibitor of surfactant phospholipid synthesis, TNFα, is overexpressed (57–63). Our analysis regarding the two hypoplastic groups created through nitrofen-exposed CDH rat model, demonstrated SP-C overexpressed in bronchi at E19.5 and E21.5 in NF versus normal lungs, whereas unchanged levels are observed after CDH-induction. In addition, SP-C is higher expressed in bronchi and terminal bronchiole at E19.5 and E21.5 in NF than CDH or control groups. Comparing with healthy lungs, CDH and NF lungs exhibit a general upregulation of SP-C expression in BADJ at canalicular and saccular stage; and in terminal bronchiole and alveolar duct at E21.5. Previous publications demonstrated an altered ratio of alveolar epithelial cells in CDH-associated lung hypoplasia, which was related to the dedifferentiation of AEC2 into AEC1 cell (64, 65), whereas Nguyen et al. report a decrease in the number of AEC1 in CDH lungs with unchanged AEC2 population in mice at E17.5. These findings are probably due to the impossibility to distinguish the differentiated versus undifferentiated AEC2 cell profile in these models.

Collectively, we describe different epithelial cell profiles in normal, NF and CDH lungs related to distinct morphological and functional features. As such, the described cellular alterations by gestational age certainly contribute to a better understanding of the epithelial profile in CDH fetuses and suggest a more careful analysis regarding the differentiated versus undifferentiated epithelial cell profiles in hypoplasia.

## Data Availability Statement

The raw data supporting the conclusions of this article will be made available by the authors, without undue reservation.

## Ethics Statement

The animal study was reviewed and approved by Direção Geral de Alimentação e Veterinária (approval no. DGAV 021328).

## Author Contributions

AG and CN-S designed and conducted the research studies. AG wrote the manuscript, which was reviewed by all authors. AG, JC-P, and CN-S analyzed the data, contributed to the article, and approved the submitted version.

## Conflict of Interest

The authors declare that the research was conducted in the absence of any commercial or financial relationships that could be construed as a potential conflict of interest.

## Publisher’s Note

All claims expressed in this article are solely those of the authors and do not necessarily represent those of their affiliated organizations, or those of the publisher, the editors and the reviewers. Any product that may be evaluated in this article, or claim that may be made by its manufacturer, is not guaranteed or endorsed by the publisher.
